# Effect of an Online Mindfulness Course for Hospital Doctors During COVID-19 Pandemic on Resilience and Coping

**DOI:** 10.1177/21501319221138425

**Published:** 2022-11-29

**Authors:** Petra Hanson, Manuel Villarreal, Majid Khan, Jeremy Dale, Sailesh Sankar

**Affiliations:** 1 University Hospitals of Coventry and Warwickshire, Coventry, UK; 2 University of Warwick, Coventry, UK

**Keywords:** wellbeing, mindfulness, hospital physician, remote training, COVID-19

## Abstract

**Introduction:**

Physicians’ wellbeing is a priority to prevent increasing rates of poor mental health and burnout, exacerbated by caregiving during the COVID-19 pandemic. Structured mindfulness courses have been shown to be beneficial, but face-to-face delivery is not always feasible in the context of busy health services. Remotely delivered structured mindfulness courses could enable wider participation, particularly at time when social distancing to prevent infection transmission is necessary. Our objective was to test the feasibility of a remotely delivered structured mindfulness course for hospital doctors during the COVID-19 pandemic.

**Methods:**

This was a feasibility study run at one English hospital between January and March 2021, when COVID-19 admissions were at a high. Interested doctors participated in a 6-session remotely delivered mindfulness course. Sessions lasted 90 min and could be attended on-line or the recording watched at later time. Main outcome measures were data on interest, course attendance and engagement, together with validated psychological outcome measures at baseline and follow-up after course completion.

**Results:**

20 doctors expressed interest to participate and 16 started the course. Of these, 12 completed at least 3 sessions (median = 4); difficulty attending resulted from conflicting clinical commitments and rosters. Twelve participants completed the follow-up survey. They rated the course highly and all perceived it to have been useful, with statistically significant (*P* < .01) improvements in wellbeing and mindfulness scores. They all stated that they would recommend this course to their colleagues and most (10/12) were interested in follow-up mindfulness sessions.

**Conclusion:**

Remotely delivered structured mindfulness training for hospital doctors was feasible, but there is a need to address the difficulties that affected attendance in order to optimize accessibility and completion of such programs.

## Introduction

Doctors across the globe are reporting increasing rates of burnout and poor mental health.^1-3^ The reasons for this are varied, with changes in work environment, including increasing workload, administrative practice and decreasing autonomy recognized as some causes.^4,5^ The mental health of healthcare professionals took a further significant hit as a result of the COVID-19 pandemic,^6^ with high reported levels of stress among health care staff.^7^

Approaches to improving doctors’ mental health, including reducing burnout (a state of mental exhaustion, depersonalization, and decreased sense of personal accomplishment^8^) and stress as well as increasing resilience (ability to bounce back after a stressful event^9^) are crucial to the delivery of safe and compassionate care to patients. Interventions aimed at improving resilience among health care staff exist and include organizational level interventions (improving workplace social support, improving organizational communication), cognitive behavioral interventions, relaxation interventions, acceptance and commitment therapy, attention, and interpretation therapy or problem-solving therapy.^10^ A mixed methods systematic review found that there was a lack of evidence for selection of interventions aimed at improving resilience in health care workers during disease epidemics and suggested more research is needed in this field.^10^

Mindfulness, defined as a capacity for enhanced and sustained moment-to-moment awareness of one’s own mental and emotional state and being^11^ may be helpful in reducing burnout and improving resilience. The importance of incorporating a mindfulness and emotional intelligence training into undergraduate and postgraduate curricula has been widely recognized.^12^ Mindfulness facilitates increasing awareness and responding skillfully to mental processes that contribute to emotional distress and maladaptive behavior.^13^ Mindfulness based interventions (MBI) have been shown to improve clinicians’ wellbeing and resilience, reduce burnout, and ultimately resulted in better patient care.^14,15^ A recent systematic review highlighted that MBIs improve doctors’ wellbeing and work performance.^16^

The COVID-19 pandemic prevented most face-to-face courses for doctors from being run due to the need to minimize social interaction and the risk of infection. This created an opportunity to investigate the feasibility of delivering mindfulness interventions for doctors via an online platform in the context of a pandemic when pressures on doctors were at a high. Our objectives were to evaluate the feasibility as well as explore outcomes of a remotely delivered mindfulness practice course for hospital-based clinicians during the second wave of the COVID-19 pandemic in England. For the purposes of the study, we adapted the Mindful practice curriculum (MPC) for online delivery. The MPC is a specifically designed program of training for clinicians centered on mindfulness-based stress reduction that was designed in the USA,^17^ and been evaluated in a face-to-face program among GP trainees in the UK.^15^ MPC has never been tested among hospital-based doctors and has not been delivered online in the UK before.

## Methods

### Recruitment

All doctors, regardless of seniority or specialty, working at a major hospital in the West Midlands were invited to express interest in participating in an online course via hospital email, as well as via posting information about the course on a hospital intranet. The recruitment took place between December 2020 and January 2021. Interested doctors were asked to access a participant information sheet, complete a consent form online, and state their preferences for dates and timing of the course to maximize participation.

### Intervention

MPC is a bespoke mindfulness-based program created for clinicians. For the purpose of this study, it was delivered on a zoom platform between January and March 2021.

The course comprised 6 sessions (90 min each) with themes such as professionalism, how doctors think, witnessing suffering, medical errors, well-being and burnout, breakdowns in communication and handling conflict compassionately. Each theme was developed integrating mindfulness principles to understand them beyond its theoretical components and to facilitate concrete changes toward an integral medical practice. The face-to-face version (the detailed description of the course delivered face to face was published by Villarreal et al^15^) was adapted to an online format using chat rooms to maintain the interactive element of the course, complying with the social distancing rules and allowing doctors to join remotely. The didactic component included presentation of information and research data relevant to each theme, and contemplative practices (including guided mindfulness practice). Participants engaged in a narrative exercise in which they recalled a clinical experience related to each theme and then discussed it with the session lead and one another.

The course ran once weekly from 7 PM to 8:30 PM for 6 weeks. The sessions usually started with a short mindfulness practice followed by the didactic component and interactive part, and closed with a guided mindfulness exercise. Participants were encouraged to practice mindfulness both during clinical practice and at home.

The MPC teachers who delivered this course are experienced practicing General Practitioners who undertook mindfulness basic teacher training and additional MPC teacher training, and have more than 8 years’ teaching mindfulness training nationally and internationally.

### Data Collection and Analysis

All participants were given a unique identifiable number, allowing pairing of their responses from pre- and post-course data. The Warwick-Edinburgh Mental Wellbeing Scale (WEMWBS) was used to measure mental well-being,^18^ resilience was measured with Smith’s Brief Resilience Scale,^19^ burnout was measured with the Oldenburg Burnout Inventory,^20^ stress was measured with Cohen et al’s perceived stress scale^21^ and mindfulness was assessed with Cognitive and Affective Mindfulness Scale-Revised.^22^ Detailed description of scoring and interpretation of these questionnaires can be found in a paper by Villarreal et al.^15^ Online questionnaires were completed via Qualtrics platform immediately before and immediately after the course. Additionally, participants were asked about the acceptability of the course and overall experience of the program in the post-course survey. No identifiable data was collected, except for the gender and specialty.

Participants were able to watch each session in their own time as all sessions were recorded. If a participant viewed a missed session, this was counted as session attended.

SPSS version 27 was used for data analyses. Normality of data distribution was checked with Shapiro-Wilk test and Q-Q plots. Data were analyzed with paired student *t*-test. *P* < 0.05 was considered as a statistically significant. All available data were included in the analysis. As this was a pilot study power calculation was not necessary. Feasibility was assessed by the number of doctors consenting to taking part, number of doctors starting the course and number of sessions attended by each participant.

## Results

### Baseline Characteristics and Feasibility

Out of approximately 800 doctors working at the hospital, 20 doctors (8 males and 12 females) consented to participate. Ten interested participants were consultants or specialist doctors, 5 were trainees, and 5 non-trainee junior doctors. Participants’ specialties varied from microbiology, orthopedics, emergency medicine, ITU, ophthalmology, anesthetics, internal medicine, pediatrics and neonates, radiology and teaching fellows.

Sixteen doctors started the course; 4 chose not to participate due to competing time commitments. Among the 16 course attendees there were 6 males and 10 females. Seven participants were consultants or specialist doctors, and 9 participants were junior doctors. The mean and median number of sessions attended were 3.6 and 4 respectively. Table 1 provides a detailed breakdown of numbers of participants and sessions attended. There was no difference between the mean number of sessions attended by consultants (*n* = 3.7) and junior doctors (*n* = 3.6). Recordings were watched by 4 participants (3 junior doctors and 1 consultant).

**Table 1.** Number of Sessions Attended by Participants.

Sessions attended	Number of participants
up to 2 sessions	4
3 sessions	3
4 sessions	4
5 sessions	3
6 sessions	2
1 video recording accessed	4
2 video recordings accessed	3

Four out of 16 participants did not complete pre and post course survey; hence, outcome measures were available from only 12 participants. The main reason for not attending was timing of the course, as it interfered with family time for some participants, as well as with clinical commitments. Some participants would prefer a later start time and shorter sessions. Those who initially consented to participate but did not start the course reported they would prefer the course to run during normal working hours. Only 4 participants viewed some of the sessions in their own time. It is not clear why others who missed sessions did not use these recorded videos.

### Acceptability of the Course

All 12 participants that completed the evaluation found the course useful and the majority (11/12) thought that the educational value was high. They also said that they would recommend this course to their colleagues and most (10/12) were interested in follow-up mindfulness sessions.

### Psychological Outcomes

Twelve participants completed psychological surveys; they had all attended at least 3 sessions. All data were normally distributed. The mean Warwick Edinburgh Mental Wellbeing Scale score improved from 43.3 (SD 6.7) pre-course to 48.7 (SD 4.3) post-course; *P* < .01. The mean mindfulness score was 24.6 (SD 3.6) pre-course and 26.8 (SD 3.4) post-course; *P* < .05. There was no significant change in scores for stress, resilience and burnout, as summarized in Table 2.

**Table 2.** Differences in Scores Between Baseline and Follow-up for Participants (*n* = 12).

Survey	Mean difference post-pre	SD	SE	Lower 95% CI	Upper 95% CI	Sig. (2-tailed)
WEMWBS	5.3	5.4	1.6	8.8	1.9	0.006
Resilience	1.8	3.9	1.1	4.2	−0.7	0.149
Burnout	−0.1	0.2	0.1	0.1	−0.3	0.233
Stress	−1.9	3.7	1.1	0.4	−4.2	0.097
Mindfulness	2.2	3.2	0.9	4.2	0.2	0.037

Abbreviations: CI, confidence interval; SD, standard deviation; SE, standard error. .

For the 9 participants who attended 4 or more sessions, statistically significant stress reduction of 2.9 (*P* < .05) was also observed.

### Qualitative Data

Participants reported a range of positive consequences from attending the course. For example, “always attempt to consider the others” perspective, acknowledge feelings better and allow more self-compassion’ (Participant 3) and “spend a bit more me-time” (Participant 4) as well as become “a bit kinder to myself” (Participant 11). Participants also reported that they intend to do more mindfulness and apply it to their clinical practice. Some expressed an interest for a calmer place at work in which they could meditate: “slow down and take a breath more at work. I wish there were somewhere you could easily pop to and meditate” (Participant 10).

Some found that the course had an impact on aspects of their life outside the workplace. As one stated, “The course has introduced to me the concept of mindfulness that I have tried to employ in other aspects of my life. I try to notice things more. If I am running, I tend to avoid music so I can listen to the birds and focus on my environment. I recognize that I need to consciously expel the thoughts of past and future to remain in the moment” (Participant 9).

The most enjoyable aspects of the course were interactions and discussions with their colleagues. “I enjoyed the whole group having discussions on topics. I like the directed pre-reading or self-tests to use before the sessions” (Participant 3). Additionally, participants enjoyed “Understanding the principle of mindfulness and being given the tools to implement this in life” (Participant 9), “Guided meditation, hearing from more senior doctors about their similar experiences at work” (Participant 12) and “Good themes- very relevant to medicine” (Participant 2).

Participants appreciated that the course was: “Run by medics for medics with relevant topics” (Participant 1). The main area for change was timing and length of the sessions, as well as having a hybrid approach, enabling both face-to-face and remote meetings. Participants suggested to have “Allocated mindful activities for the week, tips on mindfulness and meditation. Maybe incorporating a 5 to 7 min meditation daily (for people to do in their own time)” (Participant 15), “More time in the breakout rooms and for discussion” (Participant 1) and “More meditating” (Participant 10).

## Discussion

### Summary

This study demonstrates the feasibility of remotely-delivered MCP course for hospital doctors, delivered during COVID-19 pandemic. Participants from a wide range of specialties and seniority found the course useful and would recommend it to their colleagues. It proved difficult to find a time and day for the delivery of the course that suited all participants, given variation in shift patterns and other commitments. This emerged as a barrier to participation that contributed to non-attendance of sessions. However, the remote delivery enabled participation of a wide range of doctors, as shown by the range of participants’ job roles, so facilitating the sharing of clinical experiences and deep reflective practice. This was one of the most valued elements of the course which may account for participants rarely viewing the videos of sessions that they had missed.

Following completion of the MCP program, those doctors who attended at least 3 out of the 6 sessions had significantly improved wellbeing and mindfulness, with those that attended at least 4 sessions having an additional significant benefit of reduced stress levels.

While the pandemic highlighted a need for remote courses to support clinician’s wellbeing became clear. We have shown before that MPC is feasible and improves psychological outcomes among GP trainees, when delivered face-to-face.^15^ However, remote delivery, as well as opening the course to all doctors, regardless of specialties and seniority, has not been done before. During the course it became obvious that discussions among colleagues from different specialties were very popular and enriching for all participants. In fact, facilitated group discussions were reported as one the most enjoyable aspects of the course. Such a sharing of concerns and personal clinical experiences was extremely valuable and, in many ways, therapeutic to all participants, in the time of great stress and uncertainty. The lack of recorded video take-up observed in our study could be partly explained by the missed discussions with peers and thus less interest in catching up on missed session. Overall, the theme of improved self-compassion was evident from the feedback.

### Strengths and Limitations

The main strength of this study was the use of an evidence-based mindfulness training program (MPC) that we had already tested with doctors undergoing general practice training (delivered in person).^15^ Validated psychological outcome measures were used to assess effect of this remote course. Another strength was its remote delivery; this was a necessary measure during the COVID-19 pandemic in spring 2021. Participants were from various specialties, as well of different seniority, improving generalizability of our findings to hospital doctors across the country. However, it is important to recognize that clinicians from different specialties face different levels and type of stress due to the varying nature of work and as such the effects of MBI may vary between different clinical contexts.

There were several limitations of this work. As a feasibility study, we did not have a control group or long-term follow-up. However, the primary outcome of this study was its feasibility and psychological outcome measures were used as exploratory variables. In a future study, a control group should be incorporated in order to reduce bias and hence demonstrate the true effect of participating in the course. Nevertheless, the rates of depression among adults in the UK remained stable between January and March 2021,^23^ which could be used as a proxy to relatively stable stress levels experienced by hospital workers.

Participants may have been more engaged than other clinicians as they opted to take part in this study. Randomization of intervention would reduce this selection bias.

Additionally, there was a relatively small number of doctors participating in the study, with timing and length of the sessions being the main barrier to attending all sessions.

Power calculation was not done as this was a feasibility study. In a future study, this would be needed informed by the effects noted in this study. Due to the nature of online platforms, the small group discussions had a shorter duration than the face-to-face course. In addition, the course facilitators had extensive experience of delivering online mindfulness courses and both had been involved in our previous study with GP trainees^15^; the extent to which the course would have been successful with others facilitating its delivery is unknown.

Additionally, non-verbal components of communication, a critical part of the interactive exercises, may have been limited in the online course compared with the face to face delivered MPC.

### Comparison with Existing Literature

Shakir et al^12^ highlighted that mindfulness and emotional intelligence training is lacking in both undergraduate and postgraduate medical education. However, in recent years there has been increasing interest in, and evidence of positive effects of, such training internationally.^3,15,16^ Digitally delivered mindfulness interventions grew in popularity during COVID-19.^24^ While the need for interventions to enhance the wellbeing of healthcare workers caring for patients during COVID-19 pandemic was particularly pressing,^25^ our study is the first to report the use of a remotely delivered guided mindfulness program for hospital staff. A recent multicenter randomized controlled trial found that unguided digital mindfulness-based self-help app reduced healthcare workers stress.^26^ Even though the effect sizes were small, significant improvement in depression, anxiety, wellbeing, mindfulness, self-compassion, and compassion for others were found.^26^ The degree of engagement in mindfulness practice during MBIs is associated with treatment outcomes^27^ and therefore future studies should capture the extent of mindfulness practice as this affects observed outcomes.

Other approaches that are under investigation include a remotely delivered course incorporating yoga and breath works for frontline healthcare staff.^28^

Participants in our study felt that a hybrid approach (both face to face and online) to delivery of the mindfulness program would be helpful. A hybrid approach to delivery of a wellbeing program was tested by Scheid et al among US pediatricians.^29^ In this yoga-based program participants could choose the way of joining the course. The study found that participants generally preferred to attend in person sessions.

A study assessing feasibility of Mindful Self-Care and Resilience program among rural doctors in Australia found that a hybrid approach of delivery was feasible, but the number of participating doctors was only 13, with 7 finishing the 7 h course (4 h face-to-face and 3 h remote) and completing full evaluation.^30^ The rate of drop out was similar to that observed in our study, with time constraint being the significant barrier to attendance of the full course.^30^ This is similar to a study by Shapiro et al^31^ that found 8 out of 18 healthcare professionals did not engage with MBI, due to reasons such as high workplace demands or family commitments.

The variety of MBI for health care professionals impacts on generalizability of research findings as both the length and content of MBI differ. In a study by Goodman and Schorling^32^ Mindfulness Based Stress Reduction delivered as 2.5 h sessions for 8 weeks resulted in a significant improvement in burnout and mental wellbeing among healthcare providers. On the other hand, the outcomes were mixed among doctors participating in two or three 1 h long mindfulness based resilience activities, with females and more junior doctors experiencing some improvement in anxiety scores.^33^

The well-known limitations of MBI for healthcare professionals include small numbers of participants and high drop-out rates^14^ and therefore it has been recommended that MBI should be offered as voluntary modules for doctors with specific well-being needs or interest for professional development.^16^ However, given the potential of MBI for improvement of personal wellbeing, as well as the consequent effect on patient care, robust studies are needed to further define the benefits mindfulness offers to medical professionals particularly in the context of major healthcare emergencies such as the COVID-19 pandemic.

The variety of MBI for health care professionals impacts on the generalizability of research findings as both the length and content of MBI differ. In a study by Goodman and Schorling^32^ Mindfulness Based Stress Reduction delivered as 2.5 h sessions for 8 weeks resulted in a significant improvement in burnout and mental wellbeing among healthcare providers. On the other hand, the outcomes were mixed among doctors participating in two or three 1 h long mindfulness based resilience activities, with females and more junior doctors experiencing some improvement in anxiety scores.^33^

A standardized MBI should be used in future research as this will facilitate creating a robust evidence base for its application among health care workers.

### Implications for Post Pandemic Era

The impact of COVID-19 pandemic is likely to have a long-lasting effect on healthcare workers. It has worsened existing problems with burnout and depression among staff.^34^ Mindfulness interventions provide a tool for clinicians which can be beneficial both personally and professionally. In this study, we showed that online delivery of MPC course is feasible in the context of the constraints for training that existed during the first year of the COVID-19 pandemic. However, as we saw in our study, it was not possible to find a time of the day or week that suited all potential participants given variation in rotas and personal commitments. Future courses could be offered in both working hours and evenings, utilizing a hybrid approach that facilitates flexibility and participation, while maximizing the personal sharing of experience and interaction that emerged as being of particular value.

While this feasibility study identified positive effects on wellbeing and mindfulness levels, there is a need for a larger scale evaluation to understand more about the program’s take-up, completion, effects and the longer-term benefits to participants, their colleagues and their patients, as well as other psychological measures.

## Conclusion

This is the first study to show that a remotely delivered Mindful Practice Curriculum course is feasible among hospital-based doctors. In the context of the COVID-19 pandemic, improved wellbeing and mindfulness levels were observed in those who completed at least half of its 6 sessions. Given the pressures on doctors, flexibility around times of attendance with provision of hybrid courses, as well as ringfencing time to attend such training during working hours, is needed to increase access to and completion of future courses. Larger scale study of this course is needed to establish the longer-term benefits of the program.
